# Emotional Theory of Rationality

**DOI:** 10.3389/fnint.2019.00011

**Published:** 2019-04-05

**Authors:** Mario Garcés, Lucila Finkel

**Affiliations:** ^1^Department of Emotion, Cognition and Behavior Research, DAXNATUR S.L., Majadahonda, Spain; ^2^Department of Sociology, Methodology and Theory, Universidad Complutense de Madrid, Madrid, Spain

**Keywords:** emotions, cognition, evolution, behavior, consciousness, reality distortion, decision-making

## Abstract

In recent decades, the existence of a close relationship between emotional phenomena and rational processes has certainly been established, yet there is still no unified definition or effective model to describe them. To advance our understanding of the mechanisms governing the behavior of living beings, we must integrate multiple theories, experiments, and models from both fields. In this article we propose a new theoretical framework that allows integrating and understanding the emotion–cognition duality, from a functional point of view. Based on evolutionary principles, our reasoning adds to the definition and understanding of emotion, justifying its origin, explaining its mission and dynamics, and linking it to higher cognitive processes, mainly with attention, cognition, decision-making, and consciousness. According to our theory, emotions are the mechanism for brain function optimization, aside from the contingency and stimuli prioritization system. As a result of this approach, we have developed a dynamic systems-level model capable of providing plausible explanations for certain psychological and behavioral phenomena and establishing a new framework for the scientific definition of some fundamental psychological terms.

## Introduction

What is the relationship between emotion and cognition? If emotions have been historically considered as a “noisy interference” for cognitive processes (Simon, 1967), why does then emotions even exist?

Much scientific research has addressed the different areas and capabilities of the nervous system. Most of those research lines have been focused on developing models able to explain the brain’s cognitive capacities, together with its structure and dynamics at different levels (for a review see Kriegeskorte and Douglas, 2018). On the other side, emotions long stayed out of the neuroscience focus, like a collateral effect that had no easy fit within those cognitive models.

However, since the last decades of the past century, an intense debate has been active about the function and the primacy of emotion or cognition in the mental processes (Lazarus, 1984; Zajonc, 1984). These two highly polarized positions made impossible to state which of them was correct, or what was the relationship among emotion and cognition, as many necessary reasoning elements to integrate them were left apart. Wider approaches have tried to integrate both into a complete scheme (Leventhal and Scherer, 1987; de Houwer and Hermans, 2010; Gross and Barrett, 2011; Damasio and Carvalho, 2013; Li et al., 2014; Scherer and Moors, 2019), some of which have become widely spread (Moors et al., 2013), and some have been even formalized (Hudlicka, 2017; Cominelli et al., 2018). Others have also tried to derive the emotion-cognition structure from a more physiological approach (Pessoa and Adolphs, 2010; Yang et al., 2014) But until now, the exact matching between emotion and cognition has not actually been completely solved.

The main problem for the proposed models to achieve that goal is that they must clearly explain not only the dynamics of emotion-cognition interaction for the most standard behaviors but also for the most extreme ones, such as reality distortion that occurs in many pathologies like in anorexia nervosa (e.g., Body Dysmorphic Disorders). Trying to explain those extreme psychological phenomena forces the models to their limits, highlighting their structural and functional lacks and inconsistencies. Until date, none of those functional models have been able to clearly explain such phenomena from an emotion-cognition paradigm.

Finding new routes to move forward sometimes entails taking a step back and following another perspective hitherto unexplored. The numerous structures, networks, and functional levels involved in the study of the human brain require us to take that step, seek more general principles to facilitate the integration of all those elements, and deduce important implications that would otherwise go unnoticed.

In this article, we reason a new architectural framework that, while making use of simple and commonsensical elements already explored, we combine them in a different structural design, thus introducing emotions and attention as a segmentation mechanism in the information processing structure, to add to the understanding of how brain operations are optimized. This framework gives support to a new functional model which can clearly explain the existence and persistence of those extreme non-adaptive or even anti-adaptive behaviors, together with the more standard ones.

The article is divided into two complementary sections that describe the full reasoning behind the proposed model, its functional structure, and dynamics.

In the first section, we use evolutionary reasoning to find general hierarchical principles that allow us to justify the features of the nervous system and the key variables that determine the quality of its operation. We analyze the interdependence between these variables, justifying the automaticity process, and the existence of three different levels of response. We then reason the existence of intrinsic resource limitations in the system and how these limitations give rise to the attentional mechanism. From this perspective, we define the concept and role of emotions and how they control and optimize the activation and operation of advanced cognitive mechanisms.

In the second section, we analyze the structure and dynamics of the model and the interactions that occur between its different functional elements. Later, we analyze the spectrum of possible cognitive responses and how they can operate over different functional elements of the model, thus leading to different behaviors and psychological phenomena.

In this article, we explore the set of possible cognitive responses, rather than cognitive mechanisms because it is beyond the scope and length of this work and will be addressed specifically in a future article.

## Evolution

### Physics and Evolution

Why does a living being relate to its environment? If we want to understand the functioning of the nervous system that enables living beings to adapt and respond to changes, we must begin by asking this question. The fundamental physical laws are therefore the starting point of our evolutionary analysis; the second law of thermodynamics requires living beings to exchange energy and matter with their environment at an appropriate rate to maintain the structural order inherent to life, as it is defined today (Prigogine, 1997; Kleidon et al., 2005; Martyushev and Seleznev, 2006; Michaelian, 2009).

The exchange and degradation of energy in the form of nutrients and heat, therefore, stands as a fundamental principle for the existence of life (Kooijman, 2010). From a practical standpoint, in order to maintain life, the goal of a living being should be to solve its needs at the lowest cost possible (MacArthur and Pianka, 1966; Charnov, 1976). On this basis, the higher level evolutionary models (Eldredge and Gould, 1972; Sterelny, 2001; Gould, 2002; Dawkins, 2006) explore the need for every living organism to interact with its environment in order to complete the life cycle, whether for feeding, breeding, or protection (O’Neill et al., 1989), thus avoiding predation.

The evolutionary process has selected different species (Darwin, 1859), each with specific adaptive systems, allowing them to detect the environmental conditions under which they lived, as a first step to adaptation (Barton et al., 2007). Yet being able to detect environmental conditions would be useless if the living being could not act on them, whether to change, remove, or seize, and adapt to them. We can approach the study of the development and conservation of different adaptive systems, structures, and dynamics, in accordance with general principles that will serve as the basis for our reasoning.

### Evolutionary Principles

To know the principles on which we found our modeling, we need to analyze what the different adaptive systems share among the different species that they currently own or have owned in the past (Butler and Hodos, 1996; Striedter, 2005; Abzhanov et al., 2008). To do so, we must consider that, whenever a new species appears, all inherited systems and tactics must face new conditions, environments, constraints, and requirements for survival and reproduction that will test the limits of its operating range (Badyaev, 2005). From a Darwinian point of view, we can say that, within their habitats, each species is a new experiment that tests all functional elements against the filter of natural selection (Elith and Leathwick, 2009).

If we take into account that 99% of species that have existed since the origin of life have become extinct, we can postulate that the greater the number of species and the longer they retain a certain adaptive system, the more necessary, evolved, and versatile this system should be. This argument allows us to articulate three fundamental and hierarchical principles on which we will base our reasoning.

#### Necessity Principle

To be able to adapt to certain conditions, be they environmental, ecological, sexual, or otherwise, a living organism requires a system or a set of systems capable of detecting and evaluating those conditions, and of identifying or developing one or more appropriate responses to address them, choose the best available, and most importantly, implement a response, acting on the stimulus to use, avoid, or modify it. If the individual has no such system, or exaptation (Gould and Vrba, 1982), nor the ability to adapt existing ones, and the stimulus does not disappear spontaneously, the challenge will not be met. This fact is even more evident if a change affecting a system is shared among numerous highly diverse species, as it will be exposed to very different selection conditions (Boffelli et al., 2004; Hurst, 2009): we call this principle the Necessity Principle.

#### Efficacy Principle

We can assume that the environmental conditions to which species are exposed within an ecological niche can change dramatically over evolutionary time periods, thus testing the responsiveness of different adaptive systems. If effective, that is, if species successfully resolve the situation for which they were selected, the individual survives and reproduces, and the system is conserved. If not or if they cease to be effective, individuals perish and disappear; we call this conditioning the Efficacy Principle. An example of the application of this principle may be the extinction of the large dinosaurs. After more than 150 million years, all their adaptive systems failed (ceased to be effective) when a series of dramatic global changes converged in a short period of time. On the contrary, birds, the living descendants of dinosaurs, and mammals survived.

#### Efficiency Principle

We must consider that success in survival is not defined by effectiveness alone. As we have already seen, energy is the key component to maintaining the structural order of a living being (Schneider and Sagan, 2005). All adaptive systems have an implicit energy cost. The body is thus forced to permanently devote a variable amount of resources to maintain it (McEwen and Wingfield, 2003). Throughout evolutionary periods, all the resources required to maintain different adaptive systems have not always been available. Nature will, therefore, have preserved only the most efficient: those that maintain their resolving ability with as few resources as possible (Smith, 1978; Parker and Smith, 1990; Sousa et al., 2008; Kooijman, 2010). We call this the Efficiency Principle, and it can also be observed at different scales. The importance of this principle is exemplified by the human brain’s ability to fulfill all human functions with just the power of a 25 W light bulb (Kandel, 1999).

The fact that these principles are hierarchically related makes sense of some biological inefficiencies that some species can show if taken in isolation (Wedel, 2012). Hereinafter we will apply these three hierarchical evolutionary principles to different levels and scales of the nervous system, both in the study of its functional structure and the dynamics of its operation, thus facilitating identification of the critical variables that define its quality.

### Nervous System: Critical Variables and Optimization Mechanisms

If we now focus on the nervous system, we can easily infer that responses are the last link in the processing chain to face a challenging stimulus. Responses integrate and summarize all processed information, from lower sensory levels to decision-making and behavior. The success or failure of the nervous system’s adaptive capacity ultimately depends on the quality of the responses it is capable of generating (Llinás and Roy, 2009) and the quality of the execution of those responses. We will, therefore, attempt to identify which specific variables are to be adjusted and balanced in order to maximize the quality of both.

We first reflect on the *necessity* of nervous system activation, where *need* means the generation of a response only in cases of absolute necessity. If responses were generated randomly or continuously, without mediating a need, some might be effective, but they would certainly be inefficient. In order to assess the concept of necessity, the nervous system must have an indicator in order to define when to execute a response. The variable that indicates whether or not to generate a response is the Activation Threshold, and it is defined as the minimum difference between the stimulus received and an internal reference that produce neural circuitry activation (Platkiewicz and Brette, 2010). This variable is permanently and dynamically readjusted (Lu et al., 2012). A too low threshold is inefficient, generating unnecessary responses, at great cost in time, resources, and energy. A too high threshold is not very effective, preventing individuals from reacting appropriately to important stimuli, thus putting them at risk (Nagasako et al., 2003).

The second variable that we must consider is Reaction Time. It indicates the time that elapses between sensory circuit activation by the onset of a stimulus, and the moment when the corresponding response is available and starts running (Donders, 1969; Sternberg, 1969; Meyer et al., 1988a,b; Jensen, 2006; Kosinski, 2008). We can infer that the less time the nervous system takes to choose the most suitable response (Kahneman and Tversky, 1979; Doya, 2008; Cisek and Kalaska, 2010), albeit inhibitory (Logan et al., 1984), the greater the chances of survival.

Third, it is clear that swiftly finding a response can save the life of an organism, but it is also true that Accuracy is crucial in most cases. In this sense, we define *accuracy* as the difference between the response and the best possible option to respond to a stimulus, considering that both are characterized by a set of variables, such as intensity, specificity, location, timing, sequencing, and so forth. Each of these variables has an operating range within which we can say it is effective. Thus, we say that a response is effective when the accuracy of all variables is within the range that successfully solves the triggering stimulus. For example, a tennis player is *effective* if s/he hits the ball hard enough, in the right direction, within a limited time window, in a specific spatial zone, so that it passes over the net and falls anywhere within the attacking half. We say that s/he is *accurate* if s/he also intentionally places the ball at a certain point beyond the reach of the opponent. S/He will be *precise* if s/he can consistently place the ball away from his/her opponent.

If we consider the nervous system as a specialized system for processing information to produce responses, and the quality of the responses is given by the three critical variables already identified, that evolution had selected some mechanisms to optimize these variables allowing for an improved overall performance makes sense. The very existence of the following biological mechanisms could be considered a confirmation of the importance of these three variables.

Thus, mechanisms such as Memory (Atkinson and Shiffrin, 1968; Baddeley, 1992) are able to encode, store, and quickly retrieve previously processed information, making it suitable for being efficiently incorporated in new processes and reused. Pattern Recognition enables information to be shared, encoding it with fewer connections, thereby saving resources (Attwell and Laughlin, 2001) more quickly and perhaps reusing ready-made responses. Predictive Systems (Davidson and Wolpert, 2005; Kveraga et al., 2007; Bar, 2011) can recognize patterns that occur at different points in time or in sequences that, according to our reasoning, are closely related to memory capacity (Hassabis et al., 2007). Feedforward uses a prediction from predictive systems and is able to activate in advance neural and physiological components of the responses, thus creating faster circuits to send activation information along the shortest paths (Chklovskii et al., 2004; Serre et al., 2007). Feedback acts as a regulating element, allowing the nervous system to dynamically adjust its operation by checking the effectiveness of its own responses and the effects they exert on the eliciting stimuli. For instance, *efferent copy* which, combined with *inverse models* (Wolpert et al., 1998), gives way to *corollary discharge* (Crapse and Sommer, 2008), allows us to explain, for example, why we cannot tickle ourselves (Blakemore et al., 2000; Wolpert and Flanagan, 2001). The Mirror System (Rizzolatti and Craighero, 2004) makes it possible to anticipate—and imitate—the actions of others (Schaal, 1999; Molenberghs et al., 2009; Monfardini et al., 2013), thus triggering advanced social interactions and behaviors (Iacoboni, 2009; Soressi et al., 2013). And Mental Imagery (Kosslyn et al., 2001) is a high-level mechanism for optimizing critical variables. If the information developed through predictive systems is re-fed through sensory circuits (Decety, 1996; Hétu et al., 2013), it can be managed as new self-generated stimuli, which in turn can elicit new responses, either neural or physiological (Milton et al., 2008; Guillot et al., 2012; MacIntyre et al., 2013). In turn, this self-generated information could form the basis of self and social interactions (Decety and Grèzes, 2006), which is a good example of an advanced system that emerges as a combination of simpler ones. Table 1 summarizes how these mechanisms improve the overall quality of the nervous system responses through an optimization of the three critical variables we have already identified.

**Table 1:** Optimization mechanisms and critical variables.

Biological mechanisms	Variables		
	Activation threshold	Reaction time	Accuracy
Memory		*Improved*	*Improved*
Pattern recognition		*Improved*	*Improved*
Predictive systems	*Improved*	*Improved*	*Improved*
Feedforward	*Improved*	*Improved*	
Feedback	*Improved*		*Improved*
Mirror system		*Improved*	*Improved*
Mental Imagery		*Improved*	*Improved*

### Automaticity

There is, however, a very important factor we should take into account. The three variables we have identified as critical to assessing the quality of nervous system responses (*activation threshold, reaction time, and accuracy*) are interdependent. When one is modified, the others will be affected by the change. If we want to improve accuracy, we need to spend more time generating and exploring more alternatives (Garrett, 1922; Hick, 1952; Wickelgren, 1977; Meyer et al., 1988a). However, if we delay, when we finally find the best response it may no longer be needed, either because the predator has devoured us, or because our potential partner has found another (Chittka et al., 2009). Also, if we reduce the reaction time, the quality of response suffers and may no longer be accurate enough to successfully resolve the stimulus that elicited it, thus becoming ineffective. If we display unnecessary responses, albeit accurate and fast, we may waste our energy and time solving insignificant problems (Missenard and Fernandez, 2011; Lan et al., 2012), thus diminishing the availability of resources to address other and more important tasks.

The interdependence between these three critical variables is an important challenge to the nervous system when facing a stimulus. It should be able to find, at any time and for each stimulus, the best possible, or available, balance between them (Paulus et al., 2009).

The best way to achieve this optimum balance would be to have, from the beginning, a specific neural circuit, already wired to provide the most accurate response in the shortest time possible, and fine-tuned to run only when really necessary. As the optimal mechanism, evolution has developed and selected it as a priority and, because of its importance, has also incorporated it at a genetic level. These kinds of circuits are known as Reflex Circuits (Purves, 2004; Barrett and Ganong, 2010). They allow living beings to deploy a first type of highly optimized responses called Innate Responses. According to this reasoning, the more responses available in the form of reflexes, the better the balance between the critical variables that define the quality, and thus the better the overall system performance.

But this raises a new problem. Given the enormous variety and variability of possible stimuli that a living being can face (also known as *Combinatorial Explosion*), it is obvious that not all responses can be genetically wired into an innate circuit (Bair, 2015). The nervous system cannot, and should not, incorporate all possible responses innately coded, but rather the mechanisms to generate them dynamically in the most flexible and rapid way (Bateson and Mameli, 2007). Responses are encoded by networks of neurons and synapses, as are the developing and neural plasticity processes (neurogenesis, synaptogenesis, LTP, LTD, neuronal apoptosis, synaptic pruning, etc.), along with the aforementioned feedback and feedforward mechanisms, which are required to dynamically create and select the fastest, most effective, and efficient networks (Raichle et al., 1994; Chechik et al., 1998; Petersen et al., 1998; Citri and Malenka, 2008; Kaiser and Peters, 2009; Tau and Peterson, 2009). In the next section, we will see how this optimization process leads to different levels of search, development, selection, and implementation of responses.

*Automaticity* is the process by which the neural pathway associated with a response reaches the optimal balance of interconnection between its elements, thus providing the best possible relationship between the three critical variables that characterize its quality (for a review see Moors and De Houwer, 2006). However, this does not mean that an automated response is the best possible response to solve a particular stimulus (Logan, 1985; Yarrow et al., 2009). When the best response available within the limitations of individual capacities in a given context is found, the neural network that encodes it is optimized to do three things: recognize the stimulative pattern, compute the response, and run it as quickly and accurately as possible (Schneider and Shiffrin, 1977; Shiffrin and Schneider, 1977, 1984). Thus, the automaticity concept refers to the response execution quality. Depending on the intrinsic characteristics of the stimulus, it will be more, less, or even not susceptible to being automated. The different degrees of automaticity give rise to skills (Hikosaka et al., 2013), habits which are defined as “sequential, repetitive, motor, or cognitive behaviors elicited by external or internal triggers that, once released, can go to completion without constant conscious oversight” (Graybiel, 2008, p. 361). The most significant characteristics of automated responses are that the sensory events almost always elicit behavior; are resistant to dual-task interference, that is, the behavior can be executed successfully while the subject is simultaneously engaged in some other demanding secondary task (Posner and Snyder, 1975; Logan, 1979); are behaviorally inflexible; and are unaffected by reward devaluation (Ashby and Crossley, 2012).

Based on this definition, we outline the *Automaticity Principle* as follows: as a result of its own mechanisms of growth and development, and in order to fully optimize their effectiveness and efficiency, the nervous system will automate, as much as possible, the new circuits and neural networks that encode a stimulus recognition, calculation, and execution of the response associated with it.

As to be expected if it were a fundamental functional mechanism, automaticity has been systematically observed in numerous studies, with different sensory, cognitive, and motor requirements, including motor skills (Poldrack et al., 2005), driving (Charlton and Starkey, 2011), reading (Logan, 1997), music reading and playing (Stewart, 2005), and typing (Shaffer, 1975). It has also been observed in learning processes that affect highly dissimilar memory systems, whether declarative or procedural (Ashby and Crossley, 2012), and also in very different species (Helton, 2008). Despite its ubiquity, the neural bases for this mechanism are not yet clear, though there is evidence that prefrontal cortex (PFC) and basal ganglia (BG), mainly the cortico-striatum-cortical loops, are intimately related to the automaticity process (Hélie et al., 2015). Thus, the two competing paradigms, automaticity as a *“Transfer of Control from the Associative Striatum to the Sensorimotor Striatum”* (Ashby et al., 2010) and automaticity as a *“Transfer of Control from the Striatum to Cortex”* (Yin and Knowlton, 2006; Belin et al., 2009) have received wide experimental support, and identified the need for future research.

**Figure 1:**
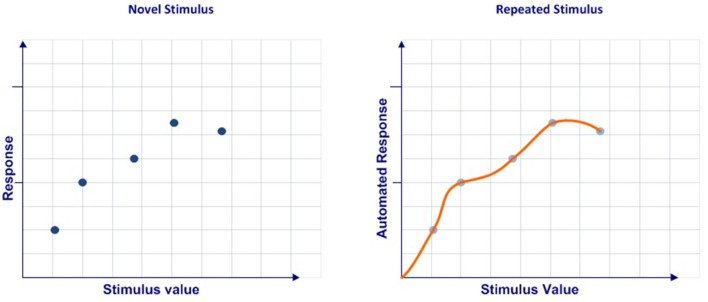
Automaticity conceptualization.

In sum, we consider that automation can be understood as a process that ranges from a discrete set of multidimensional values obtained in a limited number of cases to a continuous multidimensional function codified after a large number of events (Figure 1).

### Levels of Response

We have already seen that the first kind of responses available to the nervous system to react to stimuli are *Innate Responses* (Mameli and Bateson, 2011). We say these responses are wired because of the existence from birth of a specific neural circuitry to resolve the stimulus. The same stimulus will produce the same response. That the origin of this type of response is genetic indicates that it has been preserved by species over generations. In turn, this tells us that it has been useful in solving certain very specific, ancestral, frequent, and repetitive stimuli, including crying, coughing, pupillary dilation to changes in light, sweat secretion, heart rate control, and breathing. Within innate responses we include reflexes (Purves, 2004), Fixed Action Patterns or instincts, defined as “patterns of behavior that are fully functional from the first time they are executed, even if the individual has had no previous experience with the stimuli that elicit the response” (Alcock and Farley, 2001, p. 118).

But what happens if, because of the novelty or the variability of a stimulus, there is no innate response to enable a solution? The nervous system must develop new responses from the elements available. We call this new level of response *Cognitive Responses*. They form a broad set of more or less advanced tools which enable the body to create new solutions to address the most diverse stimuli (Quartz and Sejnowski, 1997). These mechanisms are highly flexible but have the disadvantage of requiring more time and resources to find or develop, select, and apply a response, thus reducing biological fitness. This second level of responses is useful in the absence of another effective response, or when the response time is not critical.

Once the brain finds the best possible response to a repetitive stimulus within its own capacity, it activates the *automaticity principle* in an attempt to create the most optimal pathway to process and execute the response, when necessary, as quickly and accurately as possible. This results in a third type of response we call *Automated Responses* (Raichle et al., 1994; Petersen et al., 1998).

It is important to note the difference between *innate automatic responses* and *automated responses*. While all the innate responses are genetic (Manoli et al., 2006) and therefore automatic from the outset, automated responses do not initially exist. They must first be generated through a cognitive process in the form of cognitive responses, which are later optimized, as expressed by the automaticity principle, though not all responses are susceptible to being automated.

We, therefore, define a three-level hierarchical structure for responses, starting with innate responses at the lowest level, then cognitive responses, and finishing with automated responses. Here, we summarize the three different response levels we will use from now on:

- **Innate Responses**: implemented by specific neural pathways, genetically encoded and selected to solve common situations, from an evolutionary perspective, they are highly critical for the survival of the organism. They provide automatic responses, which are very fast, accurate, and highly effective and efficient in their use of resources, selected and preserved in the inherited baggage of the species evolving over millions of years.
- **Cognitive Responses**: developed by advanced information processing systems that enable the search for new responses to face novel stimuli. Of a very different complexity, repetitive or not, they are less critical for survival. They have many different tools and multiple ways of combining them to find new solutions, but require longer analysis times and broader resource utilization, resulting in higher energy expenditure. It is important to note that cognitive responses are developed for a specific range of experienced operation and that if this range is exceeded, the cognitive response may become ineffective.
- **Automated Responses**: developed by brain optimization mechanisms that, once a new cognitive response has been found, enable the creation, selection, and pruning of the neural circuits intended to make the new cognitive response automatic. They are useful for optimizing responses to repetitive stimuli of diverse complexity, which are susceptible to being automated. Effectiveness and efficiency are reached with repeated exposure to stimuli, improving accuracy and speed as the newly created network consolidates. More time is needed to make these responses available, and the process consumes more resources.

Returning briefly to the evolutionary level, we observe that some species exhibit one, two, or all three levels of response (Table 2). Since evolution does not develop or maintain unnecessary systems, we can reason that the different levels of response emerged as a result of adaptive pressure exerted on organisms by their environment. In other words, any organism whose environmental conditions would have allowed it to survive and reproduce without problems by displaying only innate responses will not have invested resources to develop and maintain more advanced and costly brains. This explains why some animals, such as the horseshoe crab, which have survived for hundreds of millions of years without the need to strengthen its nervous system beyond a certain level of response. Another example would be sharks. In existence for around 420 million years, equivalent to seventy times the period that separates humans from chimpanzees (6–7 million years), they have not developed intelligence levels similar to humans.

**Table 2:** Types of responses by its origin and grade of automaticity.

	Genetic			
		Cognitive responses
**Non-automatic**			Problem resolution Planning Decision-making
			…
			*Automaticity process* ↓
			
**Automatic**	**Innate responses**	**Automated responses**
	Reflex	Skills
	Fixed action patterns	Habits
	Instincts Emotions	Addictions

### Response Structure

Regardless of the level to which a response may belong, it could include two types of complementary components that may or may not activate simultaneously:

- A **Physiological component** that includes autonomic and somatic systems with corresponding motor elements, endocrine, heart rate, blood pressure, etc. directed at allowing the body to perform the necessary physiological activation and physical actions to face the stimulus.
- A **strictly Neural component** that will trigger the activation or regulation of other neural networks, thus initiating new complementary brain processes.

But all these components are dynamically regulated across time, giving rise to different stages, each with its own set of active components (Dezfouli et al., 2014). Therefore, as the process it is, a response will be composed of a sequence of stages, each with its own set of physiological and/or neural components simultaneously activated (Bapi et al., 2005) and generated in different networks. Finding a new *cognitive response* is the process to identify, select, order in time, and link the appropriate sequence of stages required to face a stimulus. Automaticity is the process by which that response is wired in a new specific neural network or pathway (Dezfouli et al., 2014).

### Response Assessment

Optimization has been the keystone for all our reasoning until now; the nervous system must generate a response that effectively addresses the stimulus, only if necessary, as quickly and accurately as possible, and with the least consumption of resources. We have also seen how, to improve performance, the nervous system has developed multiple biological mechanisms in architecture and dynamics (memory, pattern recognition, predictive systems, feedback, feedforward, mirror system, automaticity) and different levels of response (innate, cognitive, and automated) that allow it to optimize the three critical variables (activation threshold, reaction time and accuracy) and their interdependence. Thus, different combinations will be optimal, depending on the context and criticality, novelty, complexity, and variability of the stimuli to be solved.

Yet all these systems and biological mechanisms would be useless if once a response is deployed the nervous system could not evaluate its success or failure. If an innate response did not effectively solve a critical stimulus, the brain would fail in its function, with the consequent risk to the organism’s survival. How does the brain evaluate the effectiveness of the responses?

Moreover, and in order to achieve the goal of optimization, there should be some mechanism to enable and disable different levels of response on demand, according to need. Before activating cognitive systems, more advanced, but also expensive and slow nervous systems attempt to face the stimuli by using faster, more accurate, and economic innate and automated response levels.

When possible, the brain uses predictive systems to generate an anticipated representation or expectation, both interoceptive and exteroceptive of the new characteristics of the stimulus once modified by its own response (Wolpert et al., 1998; Friston, 2010). Subsequently, the brain uses this expectation as a reference for comparison with the actual information received through the senses, once the response has been deployed (Blakemore et al., 2000). Importantly, this same process is also performed for neural information processed through feedback and feedforward loops without the intervention of afferent sensory and motor circuits, as in the case of thought or imagination (Phelps et al., 2001). We, therefore, consider that a response is effective if it is able to match the stimulus and expectation information. If a mismatch occurs, a specific response, if available, will be elicited. But what happens if no specific response is available because it is a novel stimulus? What if there is no expectation for comparison? And what if both match but then stop matching? What if multiple and different expectations exist for the same stimulus?

### Emotions

From an operational point of view, the best option for addressing these circumstances would be to dispose of an innate, and therefore automatic, system as quickly as possible. A system which is enabled by default to minimize the possibility of failure is capable of generating a wide range of general-purpose responses and of triggering the basic actions needed to address novel, variable, or unexpected stimuli. This system is what we call an Emotional System, and its responses are Emotions.

A stimulus could be unexpected or expected, but without an effective response to face it. There may be different expectations about the same stimulus, all with a different response availability and different degrees of efficacy. For example, a predator could, to some extent, be expected by the prey, but not so the moment or manner of attack (expectation mismatch). When the attack occurs and depending on its experientially acquired skills, the prey will or will not have a specific response (response availability). Even in the event of the prey being able to deploy a specific response, it may not be completely effective (response efficacy). Perhaps the prey can recall what happened to others in the herd, some of which were devoured, while others escaped (different expectations and responses for the same stimulus). Such casuistry, involving different functional elements throughout the information processing chain, will elicit an emotional response.

According to this reasoning, and though emotion definition is an active debate (Dixon, 2012), we now give a first partial definition of emotions as “innate contingency action programs, which act as indicators of the capacity of the already available responses to effectively resolve a triggering stimulus in a given context.”

The first two actions to be carried out by the emotional system are complementary and simultaneous, and are of two types:

- **Contingency**: displaying innate stereotypical responses to allow the individual to start managing the stimulus (e.g., fight or flight), and saving time while finding or developing a new and more specific and effective cognitive response to resolve it. This component of emotional responses has been systematically observed both in animals and humans (Lang and Davis, 2006; LeDoux, 2012).
- **Regulation**: eliciting the selective potentiation or inhibition of higher cognitive mechanisms—more advanced but slower, expensive in energy consumption and fewer—to find or develop a new, more specific, and more effective response (Kastner and Ungerleider, 2001; Pessoa et al., 2002). We develop this point in more depth when we introduce the attention topic.

It is worth noting that, unlike the model based on the concept of interruption (Simon, 1967) which considers emotions as deviations from the normal flow of information processing to cope with an unexpected event, our theory considers the emotional system as the main innate network for processing ALL sensory information, which is actively regulated only if an effective response, whether innate, cognitive, or automated, already exists. That is, if a completely novel stimulus appears, a specific response will not be available and an emotion will be elicited. This approach will be developed more thoroughly below.

### Attention

At this point, we have a new challenge to face. Through multiple afferent sensory and feedback loops, the brain permanently receives a vast amount of information from numerous stimuli, both exogenous and endogenous (Desimone and Duncan, 1995; Chica et al., 2013). In many cases these stimuli coincide or overlap in time (Fries, 2009) and must be discriminated, targeted, and/or simultaneously attended through a process of binding (Ungerleider and Bell, 2011; Bosman et al., 2012). However, the characteristics of each stimulus are different, which may increase computational requirements considerably.

To solve this problem, the nervous system could develop as much advanced circuitry as necessary, although this would contradict one of the basic principles that we stated in our initial analysis. The *efficiency principle* tells us that, as an evolutionary system, the nervous system must adjust its development and capabilities to minimize, as far as possible, the consumption of resources and time used to fulfill its function, without compromising its effectiveness.

This fact has some important effects for understanding brain dynamics:

1. The brain does not develop and even eliminates unnecessary or inefficient neural pathways (neuronal apoptosis and synaptic pruning; Low and Cheng, 2006).
2. The brain adjusts the capabilities of necessary systems, in accordance with the likely characteristics of number, frequency, complexity, variability, and simultaneity of the stimuli to be solved (plasticity).
3. Once a stimulus has been effectively resolved, and following the *efficiency principle*, the brain does not apply higher capacity mechanisms.

In addition, the *automaticity principle* tells us that once a response is found for a repetitive stimulus, and irrespective of whether it is completely or partially effective, the brain will attempt to automate execution, as a means of optimizing the balance between the critical variables that define its quality, thereby giving rise to skills, as well as to habits and addictions.

From the application of these two principles (efficiency and automaticity) we can infer, on the one hand, the relationships between the number, energy cost, and computational power of available resources and, on the other, the frequency, variability, and complexity of the stimuli the brain must solve. We can, therefore, reason that generally and throughout the evolutionary process the number of simple and repetitive stimuli is greater than complex and highly variable stimuli. We also know that many of the complex stimuli can be decomposed into simpler ones (Lerner et al., 2001; Grill-Spector, 2003; Kersten et al., 2004; Ungerleider and Bell, 2011). Thus, simple and repetitive stimuli may be managed by simpler, more numerous innate or automated pathways, allowing for parallel management. However, a more complex, novel, and heterogeneous stimuli require the joint intervention of more advanced, shortly automated, and less numerous networks in order to be solved.

This reasoning leads us to conclude that the more novel, complex, and variable the stimulus to resolve, the greater the computing power required and the lower the number of advanced networks available to carry out this function (Mani et al., 2013).

Because of this limitation of resources, conflicts often arise when accessing cognitive resources (Grossberg and Levine, 1987; Marois and Ivanoff, 2005). This justifies the need for the brain to provide a mechanism that allows it to filter and select which stimuli, at which moment, and for how long should have priority when using the advanced resources available. A classic example of interference, the color *Stroop Effect* (MacLeod, 1991; MacLeod and MacDonald, 2000), shows us what happens when two processes attempt to use the same system, in this case the verbalization system, to carry out a task, thus interfering with each other. The more automated process in daily life, in this case the reading of written text (e.g., blue), prevails over the task of verbalizing the color in which the text is written (e.g., red), a much less common task.

Yet not only are the novelty, complexity, and variability of different stimuli involved in the process. When assessing which stimuli must have priority, a fundamental variable, from an evolutionary standpoint, can make a clear difference to the survival of a living being: the Criticality of the stimulus that requests the resources. If a critical process, irrespective of its complexity and variability, does not receive priority access to the most advanced tools, the result can be fatal for the organism.

To face these problems there is a specialized set of systems (Raz and Buhle, 2006) to effectively perform the task of alerting, orienting, filtering, prioritizing, and allocating resources: attention (Norman and Shallice, 1986; Posner and Petersen, 1990; Desimone and Duncan, 1995; Petersen and Posner, 2012). The mission is to assess the various simultaneous requests to access different cognitive resources, prioritize, and allow them to optimally access those resources depending on their characteristics. But how can the attentional system evaluate the priority of different stimuli which are concurrently requiring access to cognitive systems?

According to our theory, the emotional system is responsible for assessing and qualifying all the stimuli simultaneously attempting to access cognitive resources, modulating attentional systems to prioritize and resolve conflicts, thereby assigning the available resources to different stimuli according to their criticality. This structure assigns a new role to emotions, complementary to those already described of contingency and cognitive regulation: the qualification of stimuli priority according to their own critical characteristics, simultaneity, the internal state, and current workload in the system.

Thus, we can now give a complete operational definition of what we understand as emotions, which are described as “innate contingency action programs, which act as ‘indicators’ about the capacity of the already available responses to effectively resolve a triggering stimulus in a given context, thus qualifying the stimulus’ priority and modulating attention to assign access to cognitive resources.”

By applying criticality maps (Fecteau and Munoz, 2006), the emotional system modulates attention for the different concurrent stimuli, thus creating and adjusting what we call Attentional Windows, namely, selecting and filtering a greater or lesser number of simultaneous stimuli (Vuilleumier, 2005), and allowing cognitive resources to act on them. As a result of this process, attentional windows are continually readjusted in focus and size. Therefore, stimuli can be processed in parallel and integrated if they do not require access to the same cognitive network (Pessoa, 2010), such as when we simultaneously process the image and the sound of a movie, or can be filtered, such as when we selectively eavesdrop on a conversation at a party (Fritz et al., 2007). When a stimulus is evaluated as highly critical, the attentional window is reduced and focused on the stimulus associated with it. For example, the slowing effect of temporal perception (Eagleman, 2008), tunnel vision (Godnig, 2003), and auditory exclusion, phenomena highly documented by combatants in situations of extreme stress (Artwohl, 2002; Drzewiecki, 2002). When there are no urgent requests, attention prioritizes less critical stimulus. At all times we are exposed to quantities of low criticality stimuli, both sensory and cognitive so that attention always has some information to process.

It is important to note that we have not yet introduced the notion of awareness; hence, attending to a stimulus does not mean being aware of it (Lamme, 2010). Although we will expand on this below, it is worth clarifying here that we clearly differentiate between attention (mechanism to prioritize concurrent access to shared resources), orienting (e.g., the fact of fixing gaze in a stimulus), and awareness (ability to relate the occurrence of a stimulus). In this way, we can attend to different stimuli, with or without orienting, and completely unconsciously (Armony and Vuilleumier, 2013; Capítulo 14; Pessoa, 2013; Capítulo 4). For example, in an unpublished experiment (Garcés, 2003) where subjects were rewarded when successfully detecting palindromic car plates while driving, we observed how the searching and assessment process, which initially needed to be effortful and consciously attended, gradually (in 3–4 months) became completely unattended, automatic, and unconscious. Moreover, even when the task was unrewarded, and more than 5 years after the experiment had ended, most of the subjects reported the persistence of that acquired capacity. Interestingly, all subjects also reported that, after reaching automaticity, when palindromic plates—and only those—suddenly appeared, they captured the gaze and kick-started the subjects’ consciousness, though they had had no subjective sensation of searching for them. The subjects reported a pleasant emotion when they became aware of the situation. Interestingly, many subjects report that some of the plates were numerically incorrect (not a palindromic number), but were usually morphologically very similar to correct ones (e.g., 8,838). This fact reinforces the possibility of the existence of an emotional network that works with coarse (low spatial frequency) information, before recruiting more advanced orienting and attentional resources (Vuilleumier, 2005).

Summarizing this paragraph, given the constraints the evolutionary process imposes on living beings, a limit on the number of total available resources does exist, to the extent that not all the stimuli can be processed at the same time through specific networks. Therefore, stimuli must share a set of different hub resources, recruited into dynamic networks, in order to achieve the cognitive processes needed to find or execute a response. The emotional system uses criticality maps to assess the priority of each different simultaneous stimulus, thus modulating attentional systems to manage access to cognitive systems.

### Cognitive Systems

According to our model, cognitive systems are responsible for finding new, more specific, effective, and efficient responses when those available, whether innate, cognitive, or even automated, are unable to effectively resolve a stimulus, that is, to completely match stimulus and expectation representations.

Within the category of cognitive systems, we include neural networks capable of implementing several more or less advanced ways of processing and combining them dynamically and innovatively to generate solutions of varying complexity, enabling the living being to effectively respond to stimuli. For that purpose, these systems use sensory and previously represented conceptual information (Martin, 2007; Patterson et al., 2007; Mahon and Caramazza, 2009) and their relationships, combining them to create new representations. Within cognitive systems, we include implicit learning or imitation, decision-making, working memory, logic, planning and prediction systems, theory of mind, language, imagination, and deception.

Once a stimulus gains access to cognitive systems, we must re-apply the principles of efficacy and efficiency. The brain must find an effective response while minimizing the time and resources to do so. For that purpose, it must explore the appropriate cognitive mechanism, according to the criticality and complexity of the proposed task, and only those. The existence of multiple subsystems (Norman and Bobrow, 1975; Miyake et al., 2000), with different degrees of expertise and consumption of resources and time, generates a hierarchical structure for cognitive systems that define the order in which they will go into action to solve a particular problem (Miyake et al., 2000). Several of them may sometimes operate simultaneously on the same stimulus, while at others, they will operate sequentially (Paas et al., 2004, 2010; Bapi et al., 2005).

In accordance with our reasoning, therefore, the meaning of *cognition* does not depend on the level of sophistication of the systems used to solve the stimuli, but the right balance between the problem, the constraints, the system applied, and the quality of the response, in terms of the need, effectiveness, time, and resources used to find it (Sternberg and Pretz, 2005; Neubauer and Fink, 2009; Deary et al., 2010). This will have important implications for behavior, which we will discuss below.

It is worth noting that, since cognitive responses are experientially acquired, their effective range of operation will be restricted within the limits of the aforementioned stimuli. In the event of a stimulus exceeding that range, the cognitive response could become ineffective, and the emotional response will again take control. This is, for example, the main reason why airplane pilots receive training in a wide number of improbable risky situations in flight simulators, thereby widening the range for which learned emergency responses are valid. The operative range of cognitive responses can also be extended through mental imagery pre-training, which is the case for many combatants and professional athletes (Milton et al., 2008; Guillot et al., 2012; Hétu et al., 2013; MacIntyre et al., 2013).

At the neuroanatomical level, and aligned with our reasoning about the necessity of emotional–cognitive loops, the networks approach shows that we cannot identify specific brain regions as cognitive or emotional, given that both cortical and subcortical pathways participate massively in the diffusion-integration processing of information (Swanson, 2000; Modha and Singh, 2010). Thus, some brain regions take part in numerous functions, while the same function can be executed by different regions, giving rise to conjunctural dynamic assemblies of different innate and acquired circuitry, in order to produce a behavior (Pessoa, 2014b). Accordingly, we do not differentiate between emotional and cognitive processes by the regions involved in their processing, but only on the kind of bottom-up and top-down operations that must be run on the stimulus, together with contextual and internal state information, to elicit the former or the latter (Lamme, 2003). Therefore, we move from the circuit concept to the network concept, where different necessities recruit different sets of functions that temporarily conform to a network (Pessoa, 2014c). Along these lines, Pessoa recently affirmed that “the neural basis of emotion and cognition should be viewed as governed less by properties that are intrinsic to specific sites and more by contextually determined interactions among multiple brain regions. In this sense, emotion and cognition are functionally integrated systems, namely, they more or less continuously impact each other’s operations” (Pessoa, 2014a).

As an example of our approach, both an Amazonian Indian and a city dweller will respond in the same way, with a startle reflex, when they are suddenly exposed to an unknown and unexpected loud noise. The Indian, however, will not respond in the same way as the city dweller when facing the barrel of a gun. In the first example, reflexive networks completely execute the response, while in the second case, the city dweller’s brain will recruit innate and cognitively developed representations to assess the stimulus and identify it as a threat, thus eliciting an emotional response (Phelps, 2006). A firearm is a meta-concept whose stimulative pattern must be associatively related to its killing capacities and risks through a cognitive process and, therefore, initially it has no meaning for the Indian. It is obvious that, though both stimuli—the loud noise and the firearm—may elicit a fear response from the city dweller, the networks recruited to reach that response are not the same.

### Consciousness

Although this issue goes beyond the focus of this article and we do not intend to give an explanation for the “hard problem” of what consciousness is (Blackmore, 2004), we have made an operational approach to this phenomena. In that regard, and aside from wider philosophical considerations, for the scope of this article we consider the existence of two different realities: objective reality, which represents the exogenous information that is captured and transduced through our senses, and subjective reality, which is the one we finally perceive when the sensory information has been filtered, integrated, modulated, combined, modified, and fed back through an unnumbered set of emotional, attentional, and cognitive processes. Thus, we consider that both realities are very different, supposing the first one to have a material entity. But where does the second one reside? In our brain, we suppose. Therefore, we need a system, or a set of systems, where subjective reality is finally built, whether it is localized or distributed across several networks. Our model then does not provide an explanation for what consciousness is or where it resides, but it does provide a reasoned explanation for the contents that access the conscious level, which will be described in depth when we introduce the dynamic model, given that some reasoning still needs to be outlined. Until then, consciousness will be considered as an emergent phenomenon and a final stage in information processing, which shows the results of lower levels of processing (Libet et al., 1983; Libet, 1999, 2004; Haynes and Rees, 2006; Soon et al., 2008; Haynes, 2011).

### Summary

The fundamental physical laws, together with evolutionary and adaptive processes, sustained for long periods of time, have shaped the nervous system as a highly optimized mechanism in information processing, enabling the development of responses that facilitate the effective and efficient interaction of living beings with their environment, thereby improving their chances of survival and reproduction.

As part of the optimization mechanism, due to uncertainties about the characteristics and simultaneity of stimuli that an individual will face, evolution has designated the emotional system as being responsible for carrying out three major functions:

1. Deploying broad-spectrum innate responses that allow exploration and rapidly address novel or unexpected stimuli for which there is no specific response.
2. Activating cognitive systems, responsible for the search and development of new responses, only on demand, thus improving response time and resources consumption.
3. Assessing the criticality of stimuli to be solved, modulating attention to allow priority access to the most advanced and scarce resources, if concurrency with other processes occurs.

Thus, and according to our model, the emotional system is always active and controls the dynamics of attention, which in turn regulates and prioritizes the access of stimuli information to advanced cognitive systems. Cognitive systems are able to develop cognitive responses, which in turn also modulate emotional responses, thereby closing a circular, complementary, dynamic, and interdependent architecture. According to this, emotion and cognition do not compete but collaborate, mutually complementing each other to achieve a complete and most efficient way to resolve the challenges faced by the individual.

## Functional Structure and Dynamics

### Introduction

In the first section of this article, we presented the evolutionary reasoning which allowed us to understand the fundamental role of emotions in optimizing brain function. In this section, we introduce the functional structure that emerges from this reasoning. We also analyze the dynamic model, which describes the way the various parts of functional structure interact to generate sensory, emotional, attentional, perceptual, cognitive, and behavioral phenomena.

### Pattern Recognition

As we reasoned, the first step in facing a significant stimulus is the ability to recognize the characteristics that identify it unambiguously. To that end, once sensory information is captured and encoded, one of the most important tasks of the nervous system is to recognize the emerging patterns (Scherer, 2009).

Since the nervous system does not know* a priori* which stimuli or combinations of them (patterns) will be significant, it initially needs to launch a comprehensive strategy capable of analyzing all the information it receives, especially that for which it has no innate circuits to process and respond to. A few stimulative patterns are innately represented and can be automatically recognized and processed through innate networks, such as reflexes or fixed action patterns (e.g., nipple sucking). From these innate patterns emerge more complex ones developed associatively in a dynamic learning process, thereby giving rise to capacities such as dynamic pattern recognition (e.g., text reading irrespective of the font in which it is written; Martin, 2007; Patterson et al., 2007; Mahon and Caramazza, 2009).

It is worth noting that, as significant stimulative patterns can be mono- or multimodal, and can emerge at different times and in many forms and sizes, multiple components can be recruited in parallel networks and in back and forth loops to facilitate their recognition (Martin, 2007; Pessoa and Adolphs, 2010).

**Figure 2:**
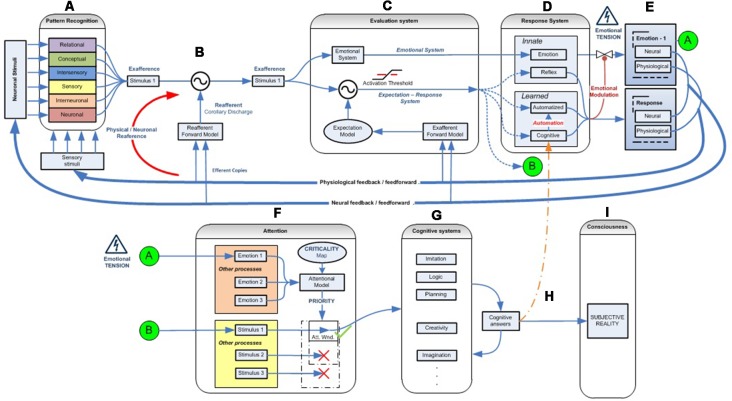
Emotional–attentional-cognitive model. **(A)** Sensory stimuli (exteroceptive and interoceptive) together with neural stimuli (information coming from diverse neural networks) are permanently received and integrated at different levels through afferent channels or internal feedback/feedforward neural pathways. **(B)** Predicted sensory consequences of nervous system’s own physiological responses are filtered. **(C)** At any given level, information is processed through two parallel systems: the emotional one (active by default) and the expectation-response system in search for a mismatch between stimulus and the expectation model. **(D)** if a mismatch is found, a response is deployed, if available. **(E)** Depending on its efficacy, the emotional circuit is partially or fully inhibited thus giving place to a variable response composed of a mix of emotional and operative components. If a specific response doesn’t exist, a full emotional response (green circle A) and the stimulus information (green circle B) are then linked and processed through attentional competition **(F)**. Depending on different parameters (number of simultaneous stimuli, their criticality, solicited resources‥.) attention will eventually give access the stimulus to cognitive systems **(G)**, where a cognitive response must be developed. **(H)** Once a totally or just partially effective response is founded, it is memorized, set as the stimulus’ by-the-moment best response and displayed, starting the automation process for trying to optimize its execution **(D)**. Also, it is made conscious **(I)**.

Through successive functional levels and recursive bottom-up and top-down loops (Lamme and Roelfsema, 2000), the nervous system gradually integrates data from the various sources of external and internal multimodal information available (Figure 2A). In parallel with the creation and optimization of a physical network, the vast initial network of neurons and connections will be optimized through the mechanisms of synaptic pruning and plasticity, reinforcing those networks by encoding functions needed to recognize and respond to significant patterns to which the living being is exposed (Johansen et al., 2014).

### Corollary Discharge

Moreover, the corollary discharge process filters and separates information concerning the stimulus from the self-generated as a part of the individual’s own response (Figure 2B). An example of this system is the inability to tickle ourselves because the brain uses the efferent motor copy to generate a proprioceptive sensory expectation (Blakemore et al., 2000). By way of example, a malfunction of this system could explain the hallucinations experienced by patients with schizophrenia. In these cases, a failure of this mechanism would prevent them from recognizing the voices they hear inside their head as generated by their own brain and from interpreting the inner voice as if it were induced by an outside agent (Ford and Mathalon, 2004).

### Emotional and Expectation-Response Systems Interaction

According to our previous reasoning, at birth, few, if any, cognitive representations or responses are available, and responses to stimuli occur through highly specific or general innate reflexive networks (McCrory et al., 2013). While innate responses are effective, the emotional response will be actively regulated. If those responses become ineffective or out of range, emotional information will regulate the innate attentional mechanisms by indicating the need to intensify the response or to search for another (Figures 2C–E). Given the limited number and range of efficacy of innate responses, as time passes and interaction with the environment and sensory experiences accumulates, it is logical to suppose that the number of episodes where innate responses become ineffective also grows, thus forcing the activation of the emotional system to regulate the development of new cognitive responses. These new cognitive responses gradually give rise to the appearance of advanced capacities, mainly cognitive, attentional, more accurate expectation generation, and more complex pattern recognition. Along these lines, we identify two complementary networks at each developmental moment.

The first is the emotional system. This innate network is always present and, by default, processes sensory information in search of emotion-laden patterns. Because these significant patterns can be either simple or complex, they can emerge at each level of information integration and processing. A hierarchical loop should, therefore, exist to systematically explore multimodal information as it gradually combines, which implies that the emotional response is always the first available, though it is not necessarily executed (Garvert et al., 2014).

The second is the expectation-response system. If innate, this network can exist from the beginning, can be acquired if cognitive or automated, or might not exist, as in the case of a novel stimulus. If it exists, it can be effective in solving the stimulus, or not (e.g., out of range).

It is important to note that both systems refer to dynamic networks formed by recruiting heterogeneous subsystems (Pessoa, 2013). This dual analysis configuration ensures that, if the stimulus is novel and/or if the available response is or becomes ineffective, the emotional response is always ready to run, without delay, deploying a stereotyped behavior that initially tackles the stimulus, while simultaneously prioritizing and regulating attentional mechanisms to compete for access to cognitive systems and find a more effective response, thus optimizing the overall functioning of the nervous system (Figure 2F).

However, as we explained in the first section when two parallel networks assume the task of solving the same stimuli, it makes no sense for both to act at the same time. For example, if both simultaneously ran two different motor actions using the same muscle groups, their effectiveness would be greatly reduced because both responses would interfere with each other (Klein et al., 2014; Morsella et al., 2016). To avoid it, both networks should be inversely connected through a modulation mechanism (Figure 2C). It stands to reason that this modulation signal should be generated from the network that implements the more specific response to solve the stimulus, the expectation-response system, to the network that displays the less specific response, the emotional one. Furthermore, because of the importance of emotional responses for the survival of living organisms, modulation must be an active process, that is, the emotional system will not be inhibited by default. Thus, the modulation of the emotional system will become an important functional subtask of specific responses, whether innate, cognitive, or automated.

The best way to clarify the role of emotions in our theory is to use, by way of example, the dead-man button: a mechanism used in trains as a safety measure to prevent accidents. The system repeatedly asks the driver to actively push a button after a random time interval. Pressing the button inhibits the action of the emergency brake, which is active by default. Thus, if the driver suffers a mishap and does not respond to the request to press the button, then there is no inhibition of the brake, which is automatically activated, stopping the train and preventing an accident. Importantly, pressing the button keeps the inhibition of the emergency system active.

**Figure 3:**
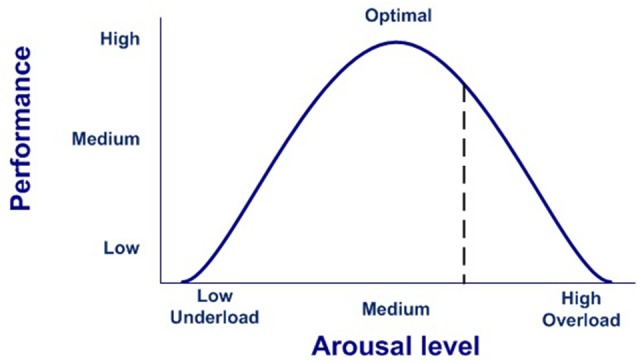
Stress curve.

In our model, the execution of a completely effective response would be equivalent to pressing the dead-man button, thereby inhibiting the activation of the emotional system, which is active by default after stimulus onset. Conversely, if there is no completely effective response, inhibition over the emotional network will not be complete, thus modulating the emotional response. In this case, emotions are the default response that mobilizes physiological and cognitive resources, but only while needed, thus optimizing the functioning of the nervous system (Bassett et al., 2009). This dynamic could account for the stress curve (Figure 3), indicating when the cognitive response is out of range, whether due to available cognitive capacity or to the intensity of the stimulus (Yerkes and Dodson, 1908; Diamond et al., 2007).

If we repeat our search for neuroanatomical and physiological correlates, we find that this mechanism has been observed at a cellular level in fear-conditioning and extinction experiments. Because the conditioned response (CR) can return after extinction under different conditions (e.g., spontaneous recovery, renewal, reinstatement, external disinhibition), conditioned memory is thought to survive the extinction process, as it is actively regulated by new memories (Maren, 2015). Therefore, not only is the CR partially depotentiated by the extinction process (LTD), it is actively inhibited in the basolateral nucleus of the amygdala (BLA), either potentiating LTP at excitatory synapses among conditioned stimulus (CS) afferents that terminate on inhibitory interneurons or limiting excitatory transmission between the BLA and central amygdala (CEA) through synaptic plasticity, induced by afferents from the ventromedial PFC (vmPFC) to inhibitory intercalated cells (ITCs), thereby suppressing the generation of learned fear responses (Quirk and Mueller, 2007; Hartley and Phelps, 2009; Furini et al., 2014; Maren, 2015). In the same vein, experiments with cognitive inhibition of fear have also shown the active inhibition of the amygdala by the dorsolateral PFC (DLPFC) through the same ventromedial vmPFC region thought to mediate the inhibition of fear response during the extinction process (Phelps et al., 2004; Delgado et al., 2008; Hartley and Phelps, 2009). This mechanism of inhibition can also be observed in startle reflex regulation when subjects are exposed to positive, neutral, and negative valence stimuli (Speed, 2012).

Thus, the neural mechanisms involved in extinction and cognitive regulation of fear seem to follow the same scheme we propose, whereby a pathway codifies the parameters of the emotional stimulus and its response, while other more specific and more effective circuitry actively modulates that response.

This model is thus consistent with more experimental models such as dual competition (Pessoa, 2009), where emotional information, whether stimulus-driven or motivational, impairs neurophysiological and behavioral responses (Yang et al., 2014). Those experimentally observed interferences are naturally explained within our model as transient states that exist in the process that leads from the completely ineffective response state (novel stimulus), where a purely emotional response is displayed, to the completely effective response state, where the emotional response is completely inhibited. Therefore, our model explains the reason for the gradual changes undergone by affective information processing, as the attentional and behavioral components are shifted from innate processing networks to more cognitively acquired and automated ones. In that sense, the impact of the emotional stimulus on behavioral impairment is not only linked to the stimulus’ level of threat (intensity, complexity, etc.), but also to the repeated experience with the stimulus (memory) and the degree of efficacy reached in solving it. Therefore, a multifactor function will once again define the set of innate and acquired resources recruited into a dynamic network to face a specific stimulus.

**Figure 4:**
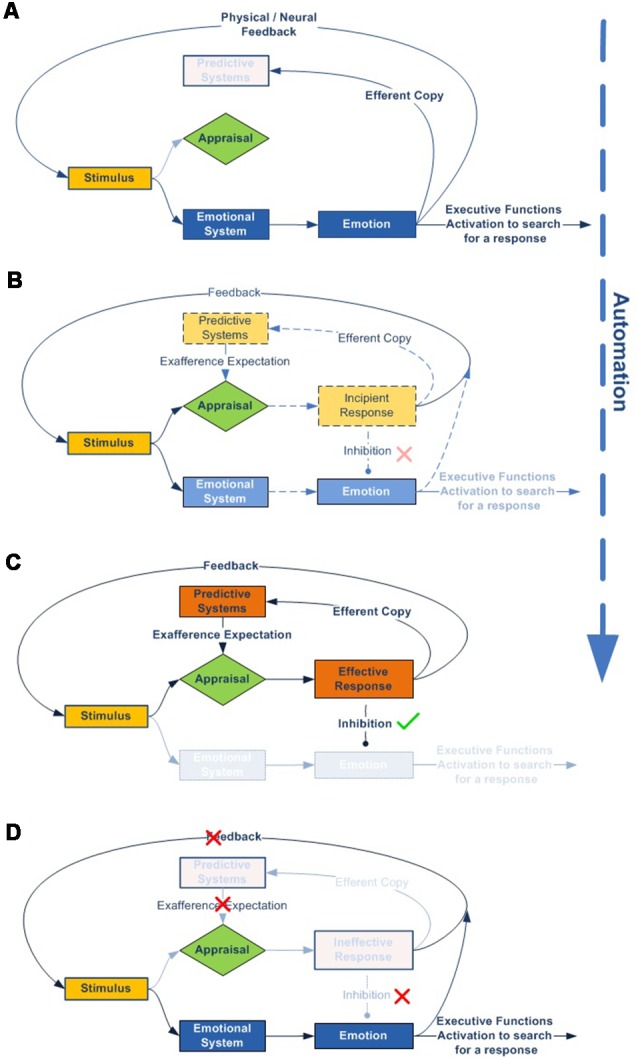
Emotional–cognitive simplified dynamic model. This figure shows the scheme for processing stimuli. **(A)** If the stimulus is novel, both for the species and the individual, there will be no innate expectation-response network. In that case, the inhibition signal for the emotional system will be disabled, which in turn will display an emotion to activate advanced cognitive systems, thus starting the search for a new and more effective response. **(B)** The emotion stays active throughout the response-search process, but is gradually inhibited as the expectation-response set becomes increasingly effective. **(C)** Once an effective expectation-response is developed, the more it is automated, or the more the predictive systems are fine-tuned for expectation generation, the more the emotion becomes unnecessary and, therefore, will be completely inhibited. **(D)** But the emotional network does not disappear; it remains inhibited as a contingency element in the event of the response becoming newly ineffective.

We can see this graphically using a simplified diagram (Figure 4):

1. According to our model, all stimuli are always simultaneously processed in parallel by two dynamic networks, the emotional and, if it exists, the expectation-response. When an organism is exposed to a novel stimulus, both for itself and for its species (Figure 4A), there is no innate specific neural circuitry to solve it, nor an expectation for comparison. Therefore, the stimulus is necessarily processed through the emotional system, by displaying an emotion. This emotional response, with its physiological and/or neural components, regulates attentional access to advanced cognitive systems in order to seek or develop a new response, while simultaneously performing more or less accurate stereotyped behavior (e.g., fight or flight).
2. As cognitive systems increasingly develop and refine an effective response to resolve the stimulus (Figure 4B), predictive systems also generate new increasingly accurate expectations for both the expected change in the characteristics of the stimulus (exaference) and the expected change in the system’s proprioceptive state (reaference; Stahl and Feigenson, 2015). This facilitates the appraisal process, which compares stimulus information as it is modified by the response and the body’s proprioceptive state, with the internally developed expectation. This structure enables the nervous system to check the effectiveness of its own responses (Friston et al., 2006).
3. In addition, as a result of repeated experience and through the automaticity process, the efficacy of the response can be gradually improved, thus inhibiting the emotional system to optimize brain function (Figure 4C).
4. But what if the response ceases to be effective? In this case, inhibition over the emotional network is again halted, thus triggering emotion as the stereotyped and best available contingency response (Figure 4D).

This model sheds light on the debate about the primacy of emotion and cognition (Zajonc, 1980, 1984; Lazarus, 1984, 1991; Leventhal and Scherer, 1987; Smith and Lazarus, 1990; Frijda, 2009; Scherer, 2009). Our model considers all these statements to be true and complementary and characteristic of the stimulus, its previous history on both the individual and the species, the context in which it occurs, and the brain optimization mechanisms which define whether the process performed on sensory information will be emotional, reflexive, cognitive, or merely an automated expectation-response.

Also, unlike the theories that consider emotion as an element that provides flexibility allowing stimuli to be isolated from responses (Lazarus, 1991), this theory regards emotions as a system optimizer and contingency response mechanism. The flexibility function is delegated to cognitive systems (cognition and metacognition), and one of the possible solutions they can deploy allows for the dynamic creation of associations, schemes, and representations (Conceptual combination, for a review see Martin, 2007; Patterson et al., 2007), which are subsequently processed by the emotional network as if they were new stimuli, in a dual bottom-up and top-down process.

Along these lines, in the 1960s, Sokolov proposed a similar model in his work on the orienting reflex and the habituation process (Sokolov, 1963; Sokolov et al., 2002). Our model re-contextualizes that work from a more global systems perspective. Thus, the gradual process of finding an effective and increasingly automatic response or the development of an increasingly precise expectation for the stimulus is known as habituation (Groves and Thompson, 1970; Thompson, 2009).

### Attentional Process

The following stage in information processing is attentional competition, which is the continuous assessment of the full set of stimulus-emotion pairs that are active at each moment and actively assigning available resources according to their criticality. Thus, as the active stimuli-emotion pairs dynamically vary their criticality, the attentional windows are refocused and expanded or narrowed to reassign access to cognitive systems.

One of the main problems when exploring the emotional regulation of attention is the fact that diverse experiments give highly disparate results: some appear to show a hard automaticity of emotional regulation over attention, while others show soft automaticity (depending on the available resources; Pessoa, 2013). Our model provides a framework to explain these different results by taking into account the definition of emotion and attention and the automaticity process we have already outlined. Thus, a set of different alternatives to attending to a stimulus can be deployed, depending once again on novelty, context, response availability, response efficacy, stimuli concurrency, criticality, and so forth. By way of example, different attentional processes can be deployed to the same stimulus (e.g., light), from a natural stimulus within a limited range (e.g., soft daylight), which is unattended and unconsciously managed through a specific reflex circuit (e.g., pupillary reflex), to a completely out-of-range stimulus (e.g., full beams at night), which is emotionally intended to be faced with spontaneous defensive behavior (e.g., raising one’s hand over one’s eyes and turning one’s head), or by deploying a cognitively learned strategy (e.g., casting one’s gaze to the side of the road).

### Dynamic Model Variables

**Figure 5:**
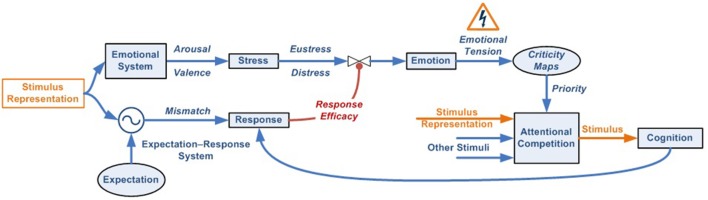
Dynamic model variables and relationships.

At this point, the relationship that arises between the different variables, functions, and factors when we explore the dynamic model is worth noting. Figure 5 provides a graphical summary.

According to this, the first variable we find is Mismatch, which indicates the degree of disagreement between stimulus and expectation representations. If the difference surpasses the activation threshold, the response, if available, is executed by the expectation-response system.

In parallel, we find the variables that characterize the emotional system (arousal and valence). Arousal refers to the level of physiological and psychological activation. Valence, in turn, indicates the approach-avoidance behavioral tendency. If we continue through the emotional system, we find Stress, which is the primary emotional response and represents a multidimensional function that combines the arousal and valence variables that give rise to distress (negative stress) or eustress (positive stress).

The next step shows the Response Efficacy (or Emotional Inhibition) parameter that, according to our reasoning, codifies the degree of inhibition the expectation-response system exerts over the emotional system, thus regulating its expression in accordance with the level of efficacy achieved. Therefore, and as we have previously outlined, we define Emotional Tension as a function that results from applying the response efficacy parameter to the stress function.

Once the emotional tension for a given stimulus is processed, it must be weighted using Criticity maps as a reference model toward assigning a priority to the stimulus representation.

Finally, through the Attentional Competition process, the brain uses the relative priority (dependent on current workload) of all concurrent processes, together with the stimuli representations, to dynamically manage shared access to the overlapping cognitive resources.

### Cognitive Responses

Once a stimulus has successfully completed the attentional competition process, it accesses cognitive systems that will be responsible for finding or developing an effective response (Figures 2G–I).

Before continuing our exploration of cognitive capacities, we should briefly contextualize our work with regard to previous definitions of coping and classifications of coping strategies identified by other authors (Lazarus, 1993), in which the term is defined as “efforts to prevent or diminish threat, harm, and loss, or to reduce associated distress. Some prefer to limit the concept of coping to voluntary responses; others include automatic and involuntary responses within the coping construct” (Carver and Connor-Smith, 2010, p. 685).

As we have explained, our model postulates that an ineffective or inexistent response to a stimulus does not inhibit the elicitation of an emotional–cognitive process responsible for seeking an effective response capable of minimizing its emotional tension. Given that in our model emotion always regulates cognition and cognition always pursues the minimization of emotion, we consider the term coping more broadly than previous authors, including not only the behaviors directed at resolving stressful or threatening stimuli but also positive and rewarding ones. For example, we consider that both a reverie about how to seduce a loved one and a rumination about the possibility of being fired at work are coping processes, with different characteristics (novelty, intensity, valence, criticality, etc.) looking to minimize their own emotional tension through the same functional structure and competing for the same cognitive resources. Using a metaphor, they are different input values for the same equation. We do not distinguish them by their focus (problem vs. emotion), volition (engagement vs. disengagement), valence (negative vs. positive), nor awareness, or even automaticity, given that, according to our model, we can deploy automated though ineffective responses (e.g., compulsive gambling; for a review, see Carver and Connor-Smith, 2010; see also habituation and sensitization below).

**Figure 6:**
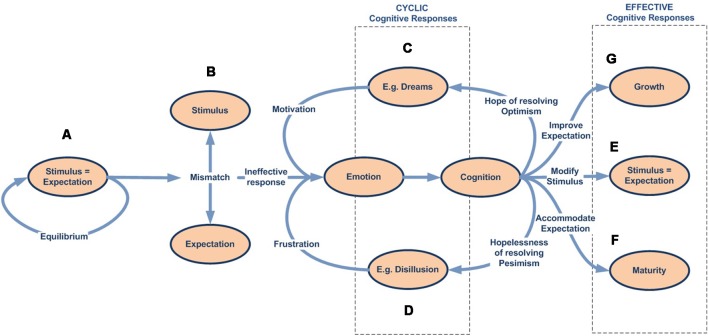
Cognitive responses. This figure shows some of the different cognitive possibilities the nervous system can find to face a stimulus. **(A)** As long as the stimulus matches the expectation, a response is not necessary. **(B)** If a mismatch occurs and an effective response is not available, an emotion is disinhibited, which in turn regulates the attentional competition to access the cognitive systems. While searching for an effective response, but not yet found, these advanced systems can **(C)** positively (optimism) or **(D)** negatively (pessimism) shape the expectation (Carver and Scheier, 1998; Carver, 2006), or alternate between both in a cyclic process (e.g., motivation and then frustration). When an effective response is finally found, it can **(E)** modify the stimulus or the associated relationship, **(F)** modify the expectation to match the stimulus, or **(G)** reframe the stimulus through a conceptual combination to create a new interpretation and therefore a new expectation about it. There are, however, other possibilities (not shown in this figure) that can modify the operation of the global system by acting upon different functional elements, thus giving rise to singular psychological and behavioral phenomena (see text in “Modify System Dynamics” for details).

Along these lines, and depending on the degree of efficacy, we identified two different groups of responses (Figure 6):

1. **Effective responses**: completely resolve the emotional tension associated with the stimulus.
2. **Cyclic responses**: the emotional tension is not fully resolved, so the emotional–cognitive process is still active, though it could remain latent if more critical processes take control of attentional resources.

This model also gives rise to three different types of cognitive responses the brain can deploy to solve stimuli:

- Responses which act on the stimulus or modify the relationship of the individual with regard to it (e.g., fight or flight).
- Responses acting on the reference model, modifying the expectation with which the stimulus is compared (e.g., acceptance).
- Responses acting on functional elements of the system, thus modifying its global dynamics (e.g., somatic silencing by anxiolytic consumption).

Furthermore, this theory considers three different areas in which cognitive responses can act:

1. **Modify the Stimulus**
  This kind of response solves the emotional tension by acting on the sensory information received. To do that it can follow two different approaches:
  (a) **Modify the stimulus to match the expectations**. In the fight or flight paradigm, the fight option would be an example of stimulus modification. This also happens when we change our physical appearance, for example, by cutting our hair or wearing makeup to achieve a (subjectively) better look.
  (b) **Modify the individual’s relationship with the stimulus**. Thus, within this category, we can include the responses of **avoidance** (e.g., flight) or approach. An example of this kind of response would be the approaching behavior small children usually show toward their mothers and the antagonistic behavior of rejection they deploy toward strangers.
2. **Modify Expectations**
  This kind of cognitive response modifies or creates new representations or new relationships between existing ones and is usually encompassed under emotion regulation capacities (Ochsner and Gross, 2014; Viviani, 2013). Under this category we can distinguish two different methods:
  (a) **Modify expectations to exactly match the stimulus**: we call this approach Maturity. Acceptance would be such a cognitive response. An example would be the process of accepting death as an inevitable and unpredictable fact of life.
  (b) **Create a new concept or find a new relationship between concepts that expand expectations (Reappraisal)**, allowing the stimulus to fit within this new framework (Martin, 2007; Patterson et al., 2007; Mahon and Caramazza, 2009; Middleton et al., 2011). We call this process Growth. An example of this type would be to consider the fact of the inevitability and unpredictability of death as elements that make life, today, here and now, something valuable and exceptional, and worthy of being intensely enjoyed. As discussed below, this process of re-contextualization of the same phenomenon is one of the best examples to ratify the emotional–cognitive structure proposed by this theory.
  Also, our model incorporates a third option that follows and allows us to explain numerous observed psychological and behavioral phenomena.
3. **Modify System Dynamics**
  Such responses can be varied and act directly on any of the functional elements of the model, thereby changing the way in which stimulus information is processed through the system. Mechanisms are numerous and include the following:
  (a) **Modify the activation threshold of a stimulative pattern**: this generates a greater or lesser activation response to the same stimulus. A simple example would be the priming effect by which the rapid presentation of a stimulus biases the subsequent response to related or unrelated stimuli (Murphy and Zajonc, 1993; Suslow et al., 2013).
  (b) **Saturate the available resources**: if we seek new and highly innovative or intense stimuli for greater criticality, they will compete with advantage in the attentional competition process and prevent other fewer priority processes from accessing cognitive systems and also consciousness. An example of this approach would be the compulsive activity and intense sensation-seeking behavior some people show after a painful breakup.
  (c) **Silence the somatic stimuli associated with the emotional response**, thus eliminating the physiological feedback that, together with neural activation, comprises the elements that define criticality, thereby minimizing attentional priority again. An example of this strategy would be the use or abuse of anxiolytics or chemicals, such as alcohol or drugs.
  (d) **Generate alternative stimuli**: one of the most fascinating collateral mechanisms postulated by this theory is the possibility of internally generating through mental imagery alternative stimuli that offer a better solution to minimizing the overall emotional tension. Thus, these imagined stimuli will compete for the same perceptual channel, outperforming the original stimulus to provide a better solution. This mechanism could be one of the foundations of multiple perceptual phenomena of reality distortion and their associated behaviors (e.g., deception). One of the most extreme areas in which we are currently conducting research is the phenomenon of body image distortion (Body Dysmorphic Disorder), which is particularly important in conditions such as anorexia nervosa (Garcés and Finkel, in preparation submitted for publication).
  (e) **Time dissociation**: changing the temporal correlation of different related stimuli can dissociate them from belonging to the same event, thereby reducing the overall emotional tension once again.

**Figure 7:**
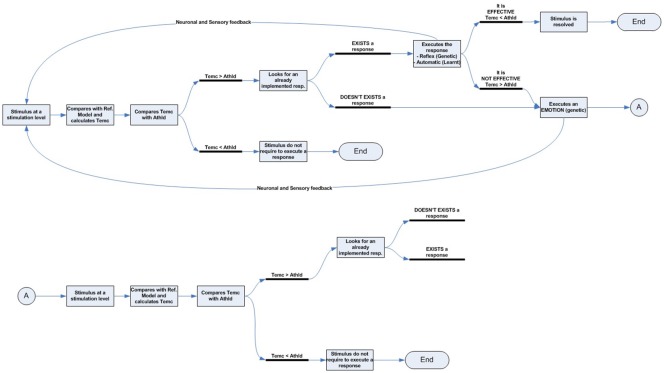
Decision-tree for emotional and cognitive responses.

As outlined above, cognitive responses are developed within a certain range, according to the characteristics, intensity, and frequency of the stimulus that elicits the search, as well as the associated emotion. If a new stimulus is beyond the range of effectiveness of a previously developed response, the emotional response is disinhibited again. Figure 7 depicts a dynamic decision-tree.

### Cognition Heuristics

To be effective in solving problems, cognitive systems must work with a number of elements and according to certain rules, as follows:

1. Cognitive system activation requires an emotional tension due to the absence of an effective response to resolve a discrepancy between the stimulus and the expectations.
2. The stimulus should have sufficient priority to be selected in the attentional competition. The more critical it is, the more time and resources allocated to process it.
3. It must have access to the various concepts and relationships that will be combined to find a new response. To manage and create a new concept, cognitive systems must find or create new relationships between previously developed concepts stored in the memory.
  As soon as a new concept or meta-concept is created, it is stored and itself becomes a new combinable element. The greater the number of conceptual elements and their relations:
  - The more flexible, advanced, and creative are the new responses.
  - The longer the process to find a new solution.
  - The greater the consumption of resources and energy.
4. Different cognitive mechanisms that can be hierarchically applied to the conceptual elements are required to find these new relationships and associations:
  - The more a cognitive system or strategy is used, the faster and more effective it will become.
  - The more advanced, the higher the quality and accuracy of their responses.
5. To find effective solutions cognitive systems need time:
  - The greater the time available to seek alternatives, the greater the number and quality of options found.
6. Emotional–cognitive processes are not disabled, unless their response not become totally effective, maintaining emotional tension even outside the attentional focus. Thus, all the unsolved stimuli remain latent until attentional competition becomes unloaded and resources are again available.

**Figure 8:**
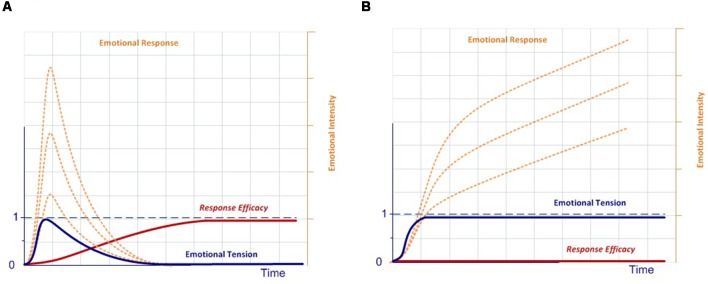
**(A)** The intensity of the emotional response is regulated by emotional tension, and this in turn by the efficacy of the response available. **(B)** When cognitive systems are unable to find a completely effective response, the only option is to increase the intensity of both: the emotional response to give greater priority to its associated stimuli in the process of attentional competition and the best response available to address the stimulus.

### Habituation and Sensitization

The intensity of the emotional response is regulated by emotional tension, and this in turn by the efficacy of the response available (Figure 8A). If cognitive systems are unable to find an effective response, the only option for the brain to cope with the stimulus is by increasing the intensity of the best response available at that time, whether emotional or specific (Silvers et al., 2015). According to our model, and to promote the search for alternatives, the intensity curve of the emotional response associated with a stimulus should follow a growing path (Figure 8B), as a mechanism to increase the criticality of the stimulus and gradually give it greater priority in the process of attentional competition. The phenomenon of sensitization can emerge as a result of this process (Groves and Thompson, 1970), which intensifies the emotional response, as well as perception, and may even completely take control before a low-intensity stimulus, a phenomenon also known as emotional hijacking.

Our model, therefore, integrates the principles reasoned by Groves and Thompson (1970), which consider the variable **S** as the activation of the emotional network and the variable **H** as the efficacy of the expectation-response network; both are related through the mechanism of inhibition postulated by Sokolov (1963).

Habituation does not occur if the response is not completely effective. The criticality of the unresolved stimulus may become gradually reduced, resulting in no access to cognitive resources, and it is being relegated, though not disabled.

### Learning, Automaticity and Control

When a new cognitive response is developed, it is stored by the new relationships between concepts and the activation timing thereof or short time potentiation (STP). The learning process together with the automaticity process will consolidate the new relationship because the stimulus occurs repeatedly. The brain always takes advantage of previous work. From an evolutionary standpoint, the efficacy principle tells us that there must always be at least one basic response to address or explore any stimulus. In other words, even when there is a new response for a stimulus, the older should be preserved. New pathways would, therefore, be a kind of short-circuit, bypassing the old response for the benefit of the new. While effective, the new responses are executed. If they fail, however, the brain gives way to the old, less effective response, while the associated emotional process reactivates the search for a new and more effective cognitive response. This approach accounts for the irrationality of many behaviors that arise when people are exposed to stimuli that exceed the range of application of already developed cognitive responses.

According to the theory and functional model outlined, control systems are themselves cognitive responses that naturally emerge as a result of using learning processes to create new relationships between cognitive responses and stimuli patterns that elicited their development.

As happens with other cognitive responses, some control responses could also be automated, thus optimizing their execution, with important implications for our attempts to understand and explain the evolution and development of behaviors, whether individual or social.

### Emotion–Cognition Systemic Dynamics

This point is one of the fundamental keys of this theory and has important implications that must be explored in order to understand psychological phenomena, behavior, and the decision-making process.

Thus far, we have focused on the dynamics of a single emotional–cognitive process that attempts to minimize its own emotional tension. But we cannot forget that all emotional–cognitive processes operate in a common space we call conceptual space, i.e., all use information stored in different memory systems to create cognitive alternatives that allow them to effectively solve their own emotional tension. This means that different processes can modify the existing conceptual relationships and create new ones within that shared conceptual space. These changes may cause other processes, whose emotional tension was inactive, to be activated or reactivated as a result of dynamically modifying already existing associations. These previously inactive processes will restart their own emotional–cognitive cycle to find a new response that will enable them to return to a minimum tension state.

At this point we must consider two things:

- According to our model, the more primary and basic a concept, the greater the number of meta-concepts and relationships built upon it (Qin et al., 2014). We can, therefore, assume that the modification of a very primary concept triggers the reactivation of more processes dependent on it, which will then generate a greater overall emotional tension in the system.
- On the other hand, we must not forget that emotional–cognitive processes do not stop until there is a fully effective response to face the stimuli that elicited them.

Considering this, the theory predicts that the brain will attempt to find the most balanced response possible for the system as a whole, i.e., that which minimizes the overall emotional tension of all the processes concerned. This means then that, in the process of assessing different options to face the same stimulus, the brain decides between them in terms of overall emotional tension associated with each possible response, including all partial tensions generated throughout the thinking chain (Kahneman and Tversky, 1979; Tversky and Kahneman, 1981, 1992).

Thus, if we take the sensations, perceptions, concepts, meta-concepts, and their associations and consider the different emotional–cognitive processes that act on them as agents competing to minimize their own emotional tension, we can postulate that the brain process of decision-making takes the form of what in game theory is known as a Nash equilibrium (Nash, 1950, 1951). This means that, once a response is found, none of the processes involved can unilaterally reduce its emotional tension by changing its own response.

By adding this new systemic level, cyclic phenomena such as sensation-seeking, altruism, or self-harmful behaviors can be explained, laying the foundations for a new paradigm in the study of motivation, decision-making, and behavior, whether in individuals or in social groups. The consequences of this theory may have important implications which we are currently researching.

### Theory Implications

This theory and its associated model allow us to put forth the hypothesis that emotions are the first and main control element for all attentional and cognitive processes and brain optimization functioning. It should be explored in more depth in future, in order to understand and scientifically explain many psychological and behavioral phenomena. The model could explain the contents that access consciousness, helping to account for the mechanisms that underlie perception and the construction of subjective reality (Garcés and Finkel, in preparation). Some of the extreme phenomena related to responding to subjective reality could also be framed within this model, mainly those where individuals respond to imagined reality by deploying extreme behaviors even contrary to their own biological fitness like in anorexia nervosa (Garcés and Finkel, in preparation). Moreover, understanding the subjective reality construction mechanisms can help to explain certain psychological concepts far unbounded, such as the construction of self-concept and self-esteem (Garcés and Finkel, in preparation), a fundamental topic given its influence on the psychological development of individuals in education and social relationships. Also, understanding the dynamics of multiple and concurrent emotional-cognitive processes could give a new standpoint to explain phenomena studied in neuroeconomics and neuromarketing, such as decision-making and the impact emotions have on them.

## Conclusion

Throughout this article, we have followed a logical reasoning to support our hypothesis that emotions are an innate resource for nervous system optimization. As such, and by default, they are responsible for managing and assessing all stimuli and regulating the activation of attentional mechanisms, which in turn prioritize access to cognitive systems and focus them to find a new and more effective response if needed. Effective responses actively regulate the expression of emotions as they become unnecessary, thus self-regulating the functioning of the system as a whole, in a bottom-up and top-down symbiotic loop. As the number of simultaneous stimuli, both exogenous and endogenous, can become numerous, and given that all operate in the same conceptual space, they can mutually influence each other, forcing the brain to find the best option among available responses to minimize the overall emotional tension. Different combinations will, therefore, be optimal, depending on the number, context, criticality, novelty, complexity, and variability of the stimuli to be solved, together with the innate or acquired capacities of the individual.

All these mechanisms enable the nervous system to deploy a wide set of different solutions, most of which are adaptive, while others are not, thereby giving rise to some more or less extreme, biased, psychological and behavioral phenomena.

## Author Contributions

Both authors listed have made a substantial, direct and intellectual contribution to the work, and approved it for publication.

## Conflict of Interest Statement

MG was employed by company DAXNATUR S.L.
